# Phytoremediation potential of *Acorus calamus* in soils co-contaminated with cadmium and polycyclic aromatic hydrocarbons

**DOI:** 10.1038/s41598-017-07831-3

**Published:** 2017-08-14

**Authors:** Nasreen Jeelani, Wen Yang, Lingqian Xu, Yajun Qiao, Shuqing An, Xin Leng

**Affiliations:** 1 School of Life Science, Nanjing University, Nanjing, 210093 P. R. China; 2 Nanjing University Ecology Research Institute of Changshu (NJUecoRICH), Changshu, 215500 Jiangsu P.R. China

## Abstract

Phytoremediation is a promising technology for the remediation of sites co-contaminated with inorganic (heavy metal) and organic pollutants. A greenhouse experiment was conducted to investigate the independent and interactive effects of cadmium (Cd) and polycyclic aromatic hydrocarbons (PAHs) on the growth of the wetland plant *Acorus calamus* and its ability to uptake, accumulate, and remove pollutants from soils. Our results showed that growth and biomass of *A. calamus* were significantly influenced by the interaction of Cd and PAHs after 60 days of growth. The combined treatment of low Cd and low PAHs increased plant biomass and Cd accumulation in plant tissues, thus enhancing Cd removal. Dissipation of PAHs from soils was not significantly influenced by Cd addition or by the presence of plants. Correlation analysis also indicated a positive relationship between residual concentrations of phenantherene and pyrene (PAHs), whereas enzyme activities (dehydrogenase and polyphenol oxidase) were negatively correlated with each other. Cluster analysis was used to evaluate the similarity between different treatments during phytoremediation of Cd and PAHs. Our results suggest that *A. calamus* might be useful for phytoremediation of co-contaminated soil.

## Introduction

Contamination with heavy metals (HMs) and polycyclic aromatic hydrocarbons (PAHs) is one of the most pressing threats to water and soil resources, as well as to human health; it can destroy natural ecosystems^1, 2^. These contaminants are often generated by various agricultural and industrial products and practices, such as chemical fertilizers, burning of fossil fuel, petroleum spills, coal tar, and residues from metalliferous mining^3, 4^. It has been estimated that 25% of global soils are highly degraded, 44% are moderately degraded, and more soil is degraded every day^5^. Soil contamination by HMs and PAHs has accelerated owing to rapid social and economic development in China through urbanization and industrialization^6, 7^. Accumulation of these pollutants in soil is becoming a major ecological concern because of their adverse effects on food safety and human health^8^. Cadmium (Cd) is considered a nonessential and potentially toxic metal and its elevated levels in soil and water present risks to human health^9–11^. PAHs are primarily formed by incomplete combustion or pyrolysis of organic matter and are deposited from the atmosphere into the soil^12^. PAHs are contamination of great concern owing to their toxic, carcinogenic, and/or mutagenic properties^13^. Thus, the remediation of soil contaminated with Cd and PAHs is urgent and imperative, requiring novel approaches to remove multiple pollutants in a cost-effective manner.

Phytoremediation is considered an eco-friendly and highly promising technology for the remediation of multiple pollutants from soil^14, 15^. The advantages of phytoremediation for soil contaminated with metal and organic contaminants have recently been investigated^16–18^. The process of remediation might be influenced by interactions of different pollutants with each other and/or with plants and the rhizosphere^19^.

Aquatic macrophytes represents a diverse group of plants with efficient capacity to remove contaminants, including HMs and (in)organic pollutants^20^. Recent studies have shown that wetland plants can potentially enhance removal and/or stabilization of metals^21^ and may also directly promote biodegradation of organic pollutants by the rhizosphere in the form of root exudates^22^ and indirectly by increasing buildup of organic carbon^23^. *Typha*, *Phragmites*, *Eichhornia*, *Azolla*, and other aquatic macrophytes are examples of wetland plants suitable for the removal of HMs^24^. *Scirpus triqueter* L. and *Juncus subsecundus* N.A. Wakef. are suitable for the removal of PAHs^25^. However, to the best of our knowledge, there have been few studies focusing on the interactions between co-contaminants on the phytoremediation abilities of these species.

Sweet flag (*Acorus calamus* L.) is a wetland, perennial, monocot plant and is considered a suitable species for wetland restoration and mitigation^26^; *it* has a great phytoremediation potential for metal-contaminated areas as it has an extensive root system, high biomass, and adaptability^7, 27, 28^. The growth and removal of pollutants by wetland plants may be influenced by the interaction between Cd and PAHs. Hence, the objectives of this study were to investigate (1) the interactive effects of Cd and PAHs on growth and development of *A. calamus*, (2) the influence of co-contaminants on uptake and removal of Cd by plants, and (3) the effect of co-contamination on dissipation of PAHs in soils.

## Results and Discussion

### Plant growth and biomass

**Figure 1:**
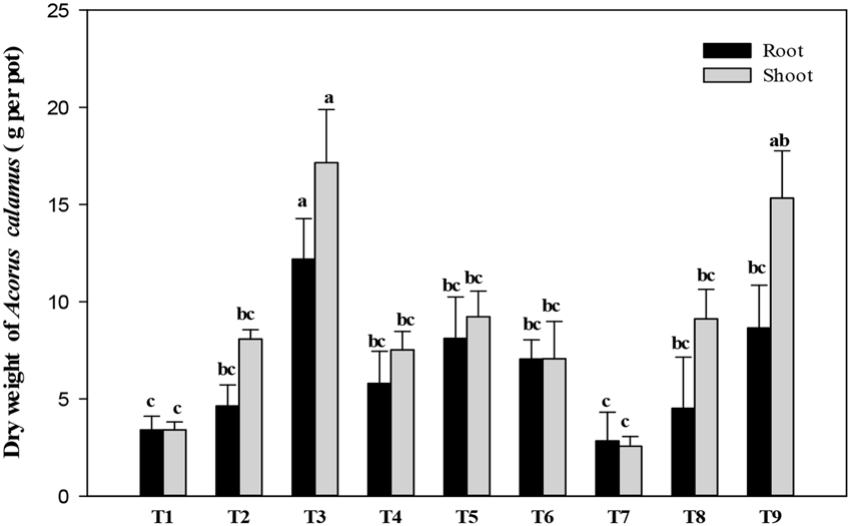
Dry weight of *Acorus calamus* was significantly influenced by polycyclic aromatic hydrocarbons (PAHs), but not by cadmium (Cd), or the interaction between the two, after 60 days of growth. Bars (mean ± SE, n = 3) with different letters (a,b,ab,bc) are significantly different based on the least significant difference test (p < 0.05). Treatments: C0P0 (T_1_), C0P1 (T_2_), C0P2 (T_3_), C1P0 (T_4_), C1P1 (T_5_), C1P2 (T_6_), C2P0 (T_7_), C2P1 (T_8_), and C2P2 (T_9_). C0, C1, and C2 represent 0, 10, and 20 mg Cd kg^−1^; P0, P1, and P2 represent 0, 50 + 25, and 100 + 50 mg PAHs kg^−1^ with PHN + PYR, respectively.

After 60 days of growth, no visible symptoms of contaminant toxicity were observed in *A. calamus* in any of the treatments. The increase in shoot and root biomass was significantly influenced by the presence of PAHs, but not by Cd or the interaction between the two (Fig. 1). Plant biomass (shoot and root dry weight) was significantly higher in high-PAH treatment alone (T_3_) as compared to that in all other treatments, except shoot biomass in high-Cd and high PAH treatment (T_9_). Compared to the low-Cd treatment alone (T_4_), biomass was higher in the combined low-Cd and low-PAH treatment (T_5_), although no significant difference was detected. However, compared to the high-Cd treatment alone (T_7_), biomass was significantly higher in the combined high-Cd and high-PAH treatment (T_9_), suggesting that a certain concentration of PAHs can increase root and shoot biomass. Although biomass of the high-Cd and high-PAH treatment (T_9_) was higher than that of the high-Cd and low-PAH treatment (T_8_), no significant difference was observed. PAHs significantly increased the biomass (root and shoot dry weight) in Cd treatments. There was no significant difference in plant biomass among the low-Cd treatments (T_4_, T_5_, and T_6_) irrespective of PAH concentrations. These results were similar to those reported by Hechmi *et al*.^29^, who found that addition of pentachlorophenol increased the biomass (shoot and root dry weight) of *Zea mays* L. in Cd treatments with 2 and 6 mg Cd kg^−1^. Zhang *et al*.^17^ reported a reduction in root and shoot dry weight of maize grown in unspiked soil while dense roots and luxuriant growth were observed in Cd- and PAH-spiked soil. Similarly, in 2012, Zhang *et al*.^30^ studied the interactive effect of Cd and PAHs on *Juncus subsecundus* and reported significant increased growth in the combined Cd and PAH treatment compared to Cd treatment alone. In the present study, *A. calamus* showed better growth in combined treatment with PAHs than without PAHs.

However, results of other studies have differed from those of the present study. For example^16^, Wang *et al*.^16^ reported that a combined treatment of Cd and Pyrene (PYR) decreased the biomass of *Kandelia obovata* Sheue, Liu & Yong, and Chigbo *et al*.^31^ investigated the effects of Cu and PYR on *Brassica juncea* (L.) Czern. and found a reduction in plant growth. These results suggest that growth responses of plants to combined contamination depend on many factors, including plant species, pollutant type, and the concentration of contaminants^18, 32^.

**Figure 2:**
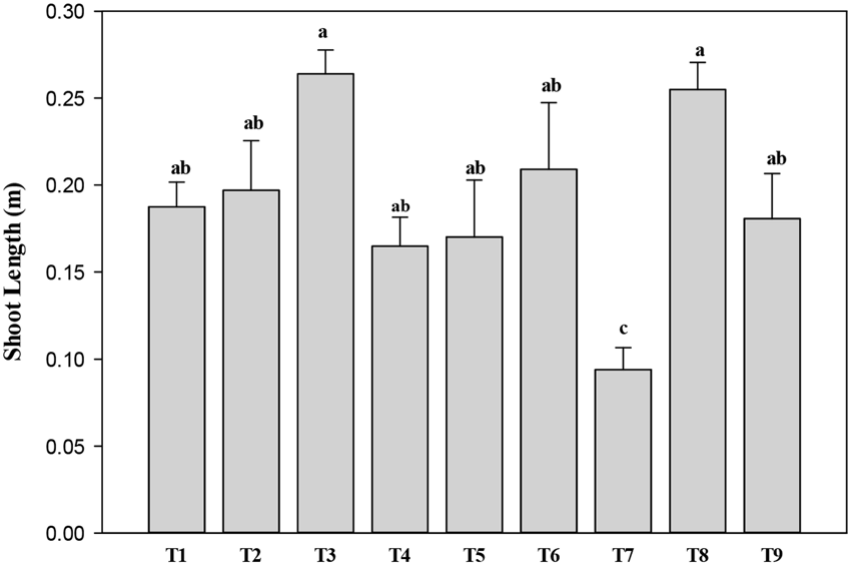
Shoot height of *Acorus calamus* was significantly influenced by polycyclic aromatic hydrocarbons (PAHs) but not by cadmium (Cd), or the interaction between the two, after 60 days of growth. Bars (mean ± SE, n = 3) with different letters are significantly different based on the least significant difference test (p ≤ 0.05). Treatments: C0P0 (T_1_), C0P1 (T_2_), C0P2 (T_3_), C1P0 (T_4_), C1P1 (T_5_), C1P2 (T_6_), C2P0 (T_7_), C2P1 (T_8_), and C2P2 (T_9_). C0, C1, and C2 represent 0, 10, and 20 mg Cd kg^−1^; P0, P1, and P2 represent 0, 50 + 25, and 100 + 50 mg PAHs kg^−1^ with PHN + PYR, respectively.

After 60 days of growth, the shoot height was significantly influenced by PAHs (p < 0.01), Cd, and the interaction between the two. Shoot height significantly increased in the combined high-Cd treatment with either low PAHs or high PAHs, compared to the high-Cd treatment (T_7_) without PAHs (Fig. 2). Compared to the high-Cd and high-PAH treatment (T_9_), shoot length increased in the high-Cd and low-PAH treatment (T_8_) but did not show any significant difference. Our results also showed that addition of PAHs significantly increased shoot height. However, Hübner *et al*.^33^ reported that an increase in the concentration of phenanthrene (PHN) decreased the shoot length of *Phalaris arundinacea* L. and *Phragmites australis* (Cav.) Trin. ex Steud.

### Cd uptake and removal by *A. calamus*

**Figure 3:**
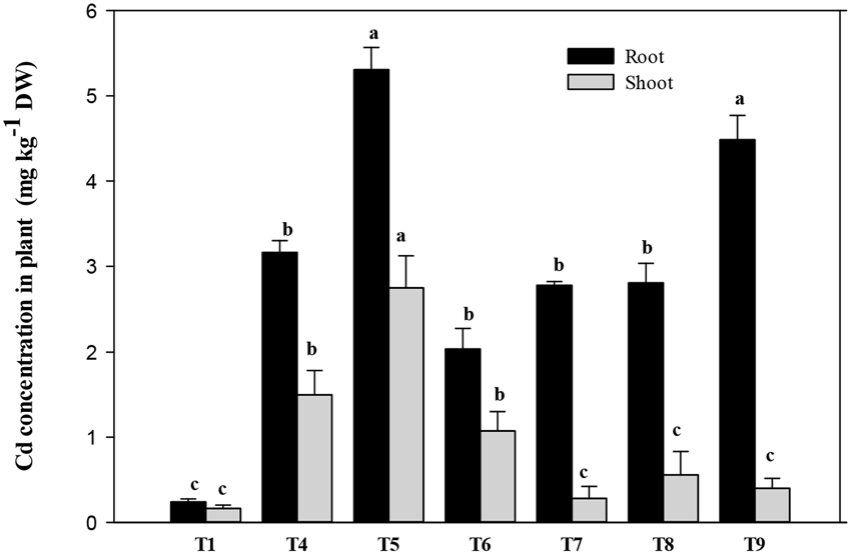
Cadmium (Cd) concentration in *Acorus calamus* was influenced by Cd, polycyclic aromatic hydrocarbons (PAHs), and the interaction between the two (p < 0.001) after 60 days of growth. Bars (mean ± SE, n = 3) with different letters (a,b,c) are significantly different based on the least significant difference test (p < 0.05). Treatments: C0P0 (T_1_), C1P0 (T_4_), C1P1 (T_5_), C1P2 (T_6_), C2P0 (T_7_), C2P1 (T_8_), and C2P2 (T_9_). C0, C1, and C2 represent 0, 10, and 20 mg Cd kg^−1^; P0, P1, and P2 represent 0, 50 + 25, and 100 + 50 mg PAHs kg^−1^ with PHN + PYR, respectively.

The concentration of Cd in *A. calamus* was significantly influenced by Cd, PAHs, and the interaction between the two (p < 0.001). The concentration of Cd in the plant tissues increased with increasing soil Cd content and increased further with the addition of PAHs (Fig. 3). Compared to the low-Cd treatment alone (T_4_), the concentration of Cd in plant tissue was significantly higher in the combined low-Cd and low-PAH treatment (T_5_) and compared to the high-Cd treatment alone (T_7_), Cd concentration in plant tissue increased significantly in the combined high-Cd and high-PAH treatment (T_9_). It is notable that irrespective of whether a soil co-contaminant was present, a higher Cd concentration was always observed in the roots. This concurs with other studies^16, 34^ that found that Cd concentration was always higher in belowground organs than in aboveground organs, irrespective of whether a soil co-contaminant was present with Cd.

**Figure 4:**
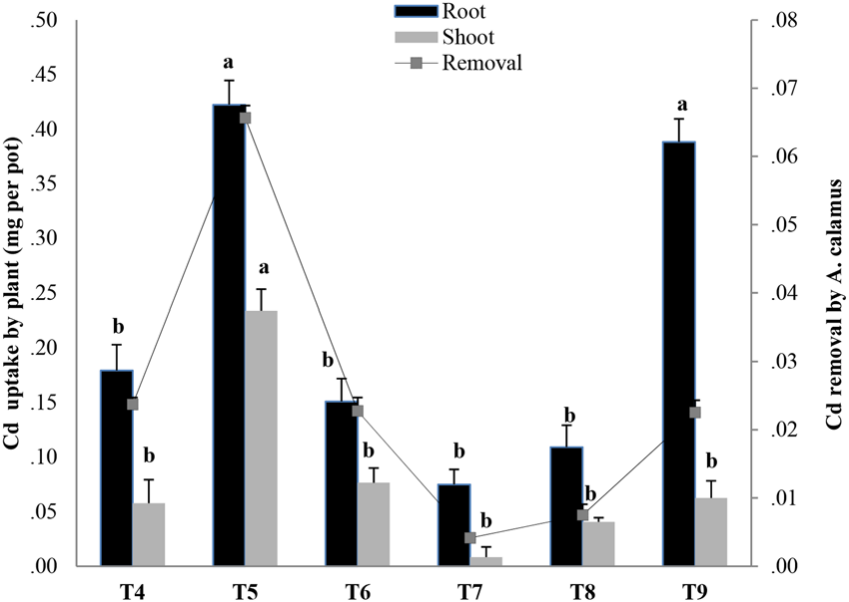
Cadmium (Cd) accumulation in plant tissues and percentage of Cd removed from soils co-contaminated with Cd and polycyclic aromatic hydrocarbons (PAHs) after 60 days of growth of *Acorus calamus*. Bars (mean ± SE, n = 3) with different letters (a,b,c) are significantly different based on the least significant difference test (p < 0.05). Treatments: C1P0 (T_4_), C1P1 (T_5_), C1P2 (T_6_), C2P0 (T_7_), C2P1 (T_8_), and C2P2 (_T9_). C0, C1, and C2 represent 0, 10, and 20 mg Cd kg^−1^; P0, P1, and P2 represent 0, 50 + 25, and 100 + 50 mg PAHs kg^−1^ with PHN + PYR, respectively.

Cd accumulation in shoots and roots of *A. calamus* across different treatments of Cd and PAHs had a similar trend to that of the Cd concentration in the plant tissues (Fig. 4). Our results showed that the roots accumulated a higher Cd concentration, irrespective of whether Cd was present alone or in a combined treatment. Metal translocation in the shoots appears to be very restricted in *A. calamus*, and it could be contended, from an ecotoxicological viewpoint, that metal transfer into shoot biomass is undesirable, as aboveground metal accumulation increases the risk of pollutants leaching or entering the food chain via herbivores and detritus feeders^35, 36^. In the present study, addition of PAHs tended to increase the uptake of Cd by the roots and decrease the phytoextraction efficiency of *A. calamus*.

Similar findings were reported by Lin *et al*.^32^, who found that co-contamination of PYR in copper-contaminated soil lessened phytoextraction capacity of Cu by *Zea mays* (maize). Zhang *et al*.^17^ also reported that Cd phytoextraction was inhibited in co-contaminated soil. However, Sun *et al*.^28^ found higher accumulation of Cd in shoots than roots of *Juncus subsecundus*; aboveground accumulation increases the phytoextraction potential of plants. Chigbo and Batty^37^ observed that co-contamination of chromium (Cr) and benzo[a]pyrene led to an increased translocation of Cr from root to shoot in *Z. mays*. These results suggest that interactions of different pollutants may exert either antagonistic or synergistic effects on bioaccumulation patterns, depending on many external and internal factors. The highest percentage removal of Cd by *A. calamus* was in the combined low-Cd and low-PAH treatment (T_5_) and was significantly higher than the low-Cd treatment alone (T_4_), but not significantly different from the high Cd-treatments.

### Bioconcentration and translocation factors

The shoot concentration factor (SCF) and root concentration factor (RCF), defined as the compartmentalized concentration ratios of Cd in plant tissues versus the soil, were used to evaluate the plant accumulation potential. The interactive effect of Cd and PAHs on the SCFs and RCFs is shown in Table 1.

**Table 1:** Concentrations of polycyclic aromatic hydrocarbons (PAHs) in plant tissues, shoot/root concentration factors (SCFs/RCFs), and the root-to-shoot transfer factors (TFs) are influenced by cadmium, PAHs, and the interaction between the two after 60 days of growth in *Acorus calamus*.

TREATMENT	SCF	RCF	TF%
T_4_	0.15 ± 0.02^b^	0.31 ± 0.03^b^	0.29 ± 0.49^a^
T_5_	0.28 ± 0.01^a^	0.53 ± 0.06^a^	0.52 ± 0.71^a^
T_6_	0.11 ± 0.07^c^	0.20 ± 0.04^b^	0.53 ± 0.10^a^
T_7_	0.01 ± 0.03^d^	0.17 ± 0.11^c^	0.22 ± 0.88^b^
T_8_	0.03 ± 0.01^d^	0.14 ± 0.05^c^	0.19 ± 0.14^b^
T_9_	0.02 ± 0.05^d^	0.22 ± 0.06^b^	0.09 ± 0.26^b^

Values (means ± SE, n = 3) followed by the same letter within columns are not significantly different according to the least significant difference test (p < 0.05). Treatments: C1P0 (T_4_), C1P1 (T_5_), C1P2 (T_6_), C2P0 (T_7_), C2P1 (T_8_), and C2P2 (T_9_). C0, C1, and C2 represent 0, 10, and 20 mg Cd kg^−1^; P0, P1, and P2 represent 0, 50 + 25, and 100 + 50 mg PAHs kg^−1^ with PHN + PYR, respectively.

The SCFs and RCFs significantly increased in the combined low-Cd and low-PAH treatment (T_5_) compared to the low-Cd treatment alone (T_4_). SCFs and RCFs significantly decreased with the addition of Cd and were influenced by Cd, PAHs, and their interaction. These results were similar to those reported by ref. 28, who found that shoot and root concentration factors decreased significantly in *Juncus subsecundus* with the addition of Cd. However, the transfer factor (TF), the ratio of shoot to root metals, was 29 in the low-Cd treatment alone (T_4_) and increased significantly in combined treatments to 52 (T_5_) and 53 (T_6_). In the high-Cd treatment alone (T_7_), the TF was 22 and decreased in combined treatments to 19 (T_8_) and 9 (T_9_). Zhang *et al*.^30^ also reported that the ratio of shoot to root transfer decreased with incremental increases in soil Cu content in *Zea mays*. Plants exhibiting TF values less than one are unsuitable for extracting metal from soils^38^. In, general wetland plants are non-hyperaccumulators that store metals in belowground tissues^39^. Hence, *A. calamus* is unsuitable for phytoextraction and can be recommended for phytostabilization of Cd-contaminated sites.

### Dissipation of PAHs from soil

**Figure 5:**
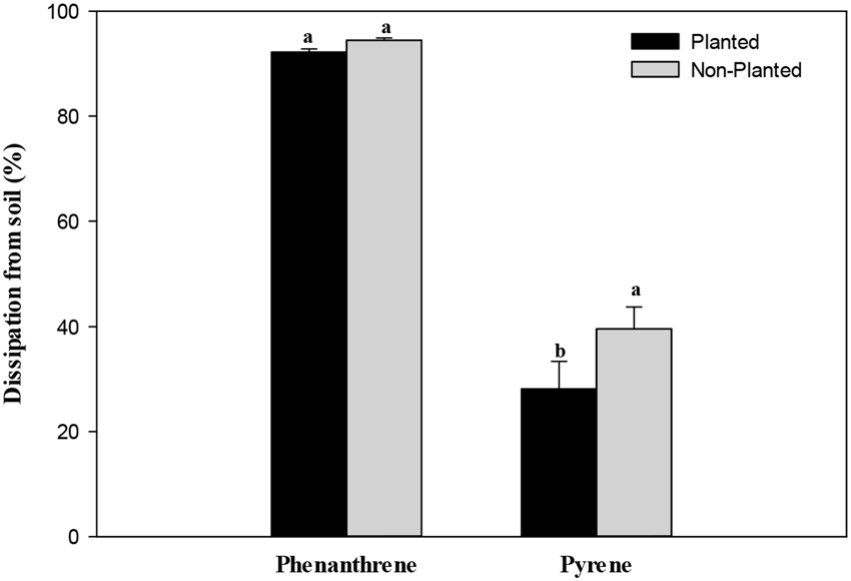
The percentage of polycyclic aromatic hydrocarbons (PAHs) dissipated (%) from soils influenced by PAH treatment after 60 days of *Acorus calamus* growth (there was no significant interaction between Cd treatments and soil with or without plants). Bars (mean ± SE, n = 18) with different letters (a,b) are significantly different based on the least significant difference test (p < 0.05).

The dissipation of PAHs was not significantly influenced by Cd addition after 60 days of plant growth. The percentage dissipation of PHN from soil was much higher than that of PYR, which concurs with other studies^16, 25^. On average, 92% and 94% of PHN dissipated in both planted and non-planted soil, whereas only 22% and 39% of PYR dissipated in the planted and non-planted soil, respectively (Fig. 5). Potential mechanisms for the dissipation of PAHs include biotransformation, biodegradation, plant uptake, and plant metabolism, or abiotic dissipation, including photodegradation, volatilization, and incorporation into soil organic material^32^. Of the four possible dissipation pathways, including abiotic losses, microbial degradation, plant uptake, and promotion from root exudates^40^, abiotic losses of PHN (80% of the initial amount) and PYR (57% of the initial amount) were the greatest. In this study, leaching of PAHs was blocked by placing filter paper inside the bottom of the pot before the experiment was started.

In the present study, the dissipation of PYR was significantly lower in planted soil than non-planted soil, which suggests that plants could accumulate such hydrophobic compounds in the rhizosphere; however, they dissipate with time^30^. However, increased degradation of PAHs in soil with plants versus soil without plants may take six months^41^. The contribution of plants to PAH dissipation is primarily based on the beneficial effect of roots^23^. Previous studies have reported a number of plant species that enhance the removal of PAHs^42, 43^ due to the increased microbial activity and degradation mediated by plant-secreted enzymes in the root zone. Plant species differed considerably in root parameters and rhizosphere characteristics^44, 45^, and the removal efficiency for PAHs may vary with species and many environmental factors^42^. Studies have reported that the presence of metals at high concentration can inhibit a broad range of microbial processes in soil^2, 46^. The high Cd and PAHs might be toxic to specific group of microorganisms, and the presence of PAHs may exasperate Cd toxicity to microorganisms^47^. Moreover, a recent study showed that climatic change can inhibit plant-microbe interactions in the rhizosphere that play a vital role in the degradation of organic contaminants^5^.

Several studies found that plant uptake and accumulation of PAHs was minimal; additionally, plants might accumulate PAHs from the air rather than from soil^18, 48^. Hence, in the present study, plant uptake and accumulation of PAHs were not determined. Further study is needed to confirm plant-microbe interactions in the co-contaminated soil.

### Correlation and cluster analyses

In the present study, correlations between different treatments and biological parameters were measured after 60 days of plant growth. The high-PAH treatment (T_3_), low-Cd and low-PAH treatment (T_5_), low-Cd and high-PAH treatment (T_6_), and high-Cd and high-PAH treatment (T_9_) were positively correlated with each other (p < 0.01, 0.05; Table 2), whereas dehydrogenase activity (DHN) and polyphenol oxidase (PO) content were positively correlated with the combined low-Cd and low-PAH treatment (T_5_) and the low-PAH treatment alone (T_2_), and negatively correlated with each other (p < 0.05). However, no correlation was found between high Cd and high PAH treatments and soil enzymes (DHN and PO). Soil enzymes are capable of decomposition of organic contaminants by catalyzing chemical reactions in soil^49^. Schooner *et al*.^50^ identified plant enzymes as the causative agents in the transformation of contaminants mixed with sediments and soil. Dehydrogenase activity in soils is very important and is a good indicator of soil microbial activity^51, 52^ because dehydrogenase occurs intracellularly in all living microbial cells^53, 54^. In the present study, the decrease in PYR removal in the soil was related to the changes in soil microbial activity, which in turn is known to be reflected by DHN and PO levels. Strong, positive correlations between dissipation rates of PAHs and soil biological parameters have frequently been observed^55–57^. Further research is needed to better understand the mechanisms of PAH dissipation and biological parameters in soils co-contaminated with metals and PAHs.

**Table 2:** Correlations between residual concentrations of PAHs and different biological parameters in planted soil (n = 18).

Treatment	PHN						PYR						DHN	PO
	T2	T5	T6	T3	T8	T9	T2	T5	T6	T3	T8	T9		
T2	1													
T5	−0.483	1												
T6	0.301	−0.980	1											
T3	−0.058	−0.846	0.934	1										
T8	0.145	−0.936	0.987	0.979	1									
T9	0.795	0.147	−0.340	−0.652	−0.485	1								
T2	0.992	−0.587	0.416	0.065	0.266	0.714	1							
T5	−0.501	1.000*	−0.976	−0.835	−0.929	0.127	−0.604	1						
T6	0.298	−0.980	1.000**	0.936	0.988	−0.343	0.413	−0.975	1					
T3	−0.058	−0.846	0.934	1.000**	0.979	−0.652	0.065	−0.835	0.936	1				
T8	0.163	0.785	−0.892	−0.995	−0.953	0.728	0.040	0.773	−0.894	−0.995	1			
T9	0.829	0.090	−0.285	−0.607	−0.434	0.998*	0.753	0.070	−0.288	−0.607	0.687	1		
DHN	−0.446	0.999*	−0.988	−0.867	−0.950	0.188	−0.553	0.998*	−0.987	−0.867	0.810	0.131	1	
PO	0.999*	−0.522	0.344	−0.013	0.190	0.767	0.997*	−0.539	0.340	−0.013	0.118	0.802	−0.153	1

PHN: Phenanthrene. PYR: Pyrene. DHN: Dehydrogenase. PO: Polyphenol oxidase. *Correlation is significant at p < 0.05. **Correlation is significant at p < 0.01.

**Figure 6:**
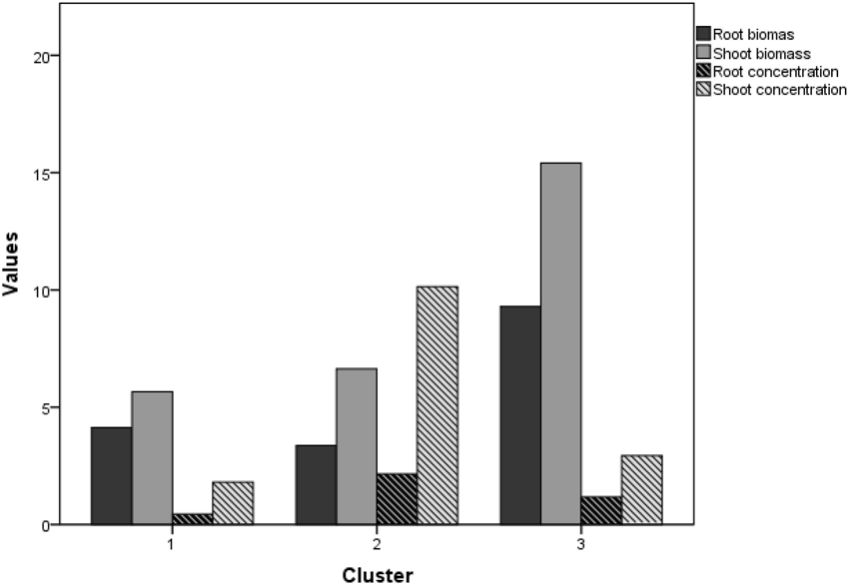
The K-means clustering analysis, with three clusters.

K-means clusters were selected as the appropriate metric for examining the combined responses of root and shoot biomass and root and shoot concentration to the different treatments (Fig. 6). In Cluster 1, the variables were found to be highly responsive to the treatment classes. About 45% of the treatments belonged to cluster 1, showing a very high response to the control (T_1_), low-PAH treatment (T_2_), low-Cd and high-PAH treatment (T_6_), and high-Cd and high-PAH treatment (T_9_).

Cluster 2 included non-responsive variables, with only 22% of treatments (low-Cd treatment (T_4_) and high-Cd treatment (T_7_), while cluster 3 had a moderate response and comprised 31% of treatments (high PAH (T_3_), low Cd and low PAH (T_5_), and high Cd and low PAH (T_8_)).

## Conclusion

Phytoremediation is a promising solution for co-contaminated sites. This study explored the phytoremediation potential of *A. calamus* in soil co-contaminated with Cd and PAHs. The addition of PAHs significantly increased biomass in plants grown in co-contaminated soil. The concentration of Cd in plant tissues was elevated in the presence of PAHs however, results indicated that Cd in *A. calamus* largely accumulated in the roots, rather than the shoots, and the TFs were <1.0 for all treatments. Cd accumulation in the roots is considered relatively stable, from the viewpoint of future release to the environment. Dissipation of PAHs from soils was not significantly influenced by Cd addition or by the presence of plants. Correlation analysis also indicated a positive relationship between various treatments of residual concentrations of PHN and PYR (PAHs), whereas enzyme activities (DHN and PO) were negatively correlated with each other. Cluster analysis revealed similarities between different treatments during phytoremediation of Cd and PAHs. From our results, it can be concluded that *A. calamus* is effective for phytoremediation of soil co-contaminated with Cd and PAHs. The high biomass production and resistance to different environmental stress make them ideal candidate for uptake and removal of pollutants from contaminated soil. Further intensive research is required to elucidate the effects of root morphology on uptake and removal of Cd and PAHs from co-contaminated soils.

## Material and Methods

### Soil treatment

Topsoil for our study was purchased from Klasmann Deilmann, Co., Germany. The main characteristics of the soil were as follows: organic matter content = 90%; pH = 6.5; EC = 35 mS·m^−1^; NPK (14:10:18) 1.0 kg m^−3^.

PAHs (phenanthrene (PHN) >97.0% purity and pyrene (PYR) >97.0% purity; Tokyo Chemical Co., Japan) were spiked into the soil at concentrations of 0 (control; P0), 50 PHN + 25 PYR (low; P1), or 100 PHN + 50 PYR mg kg^−1^ (high; P2). PHN and PYR were dissolved in 25 ml of acetone for each treatment and in the control and added to 25% (by weight) of the soil. The acetone was evaporated off in a fume hood, and the treated soil for each sample was mixed three times with the remaining 75% of untreated soil, following an established protocol^58^.

Cd (as CdCl_2_ × 2.5 H_2_O, analytical grade; Nanjing Chemicals) was dissolved in Milli-Q water and added to the PAH-spiked soils at concentrations of 0 (control; C0), 10, (low; C1), or 20 mg kg^−1^ (high; C2), resulting in a total of nine treatments including C0P0 (T_1_), C0P1 (T_2_), C0P2 (T_3_), C1P0 (T_4_), C1P1 (T_5_), C1P2 (T_6_), C2P0 (T_7_), C2P1 (T_8_), and C2P2 (T_9_). The spiked soil was then packed into pots (1 kg soil per pot) and equilibrated in a greenhouse for 2 days before being used for planting.

### Planting

The experiment comprised a randomized design, with nine treatments and three replicates per treatment. Plastic pots of 21.5 cm height were used, and a piece of filter paper was used to line the bottom to prevent PAHs from leaching out of the soil. Pots spiked with PAH included a ‘no-plant’ treatment to calculate reduction in PAHs without plants. One kilogram of each spiked soil was placed in each pot. One healthy, two-week-old seedling of *A. calamus*, sized 4–6 cm (purchased from Suqian Flower Company, China), was planted in each pot. A plastic tray was placed under each pot to prevent PAHs from leaching out of the soil. Pots were irrigated every day with 20 ml deionized water to maintain the soil moisture during plant growth. The experiment pots were periodically repositioned to minimize edge effects on plant growth.

### Sampling and measurements

After plant establishment, aboveground growth (shoot length) was measured weekly and plants were harvested after 60 days of growth. Plant shoots and roots were carefully washed several times with distilled water to remove adhering soil particles. All samples were dried to a constant weight at 70 °C for 5 days in a forced air cabinet, weighed for dry weight (DW) biomass, and ground to powder (<0.25 mm) prior to Cd analysis. The soil from each pot was homogenized and divided into two samples. One sample was stored at 4 °C prior to analysis of enzymatic activity. The second sample was freeze dried, ground to powder (<0.25 mm), and stored at −80 °C before PAH analysis.

### Analysis of plant and soil samples

Cd concentrations were determined using a mass spectrometer (ICP-MS 7700x, Agilent Technologies, USA) after digesting plant material in a heated mixture of concentrated nitric and perchloric acid. The shoot concentration factor (SCF) and root concentration factor (RCF) were calculated as the Cd concentration in shoot or root divided by the Cd concentration in soil. The translocation factor (TF) was calculated as the shoot/root Cd concentration.

Phenanthrene and PYR were analyzed according to a previously reported procedure^48^. Two grams of soil was mixed with 10 ml dichloromethane in a glass centrifuge tube, and samples were ultrasonicated for 1 h and then centrifuged at 3,000 × *g* for 10 min. Then, 3 ml of the supernatant was filtered through a 2 g silica gel column with a 15 ml hexane and dichloromethane mixture (1:1, v/v). The solvent fraction was then evaporated, and the residue was dissolved in methanol for a final volume of 5 ml. The extract was then filtered through a 0.22 μm filter, and samples were analyzed for PAH concentrations with a high-performance liquid chromatography system fitted with a 4.6 × 250 mm reverse phase C_18_ column, with methanol/water as the mobile phase at a flow rate of 1 ml/min. Chromatography was performed at 30 °C. The excitation wavelengths of PHN and PYR were detected at 243 and 235 nm using a UV detector, respectively. The average recoveries of PHN and PYR obtained from spiked soil samples with known concentrations were 91.2% (n = 4, RSD < 2.14%) and 94.5% (n = 4, RSD < 1.98%), respectively.

Soil dehydrogenase activity was measured by the reduction of 2,3,5-triphenyltetrazolium chloride to 1,3,5-triphenylformazan, as described previously^42^. Soil polyphenol oxidase activity was measured according to the methods described earlier^59^.

### Statistical analysis

Two-way analysis of variance was used to compare the effects of Cd, PAHs, and their interaction on plant growth, biomass, pollutant concentration, and accumulation. We also examined the TF in plant tissue, Cd removal by plants, and PAH dissipation from soils. All data were statistically analyzed using SPSS 19.0 and the least significant difference test was applied to identify significant differences between the means. The PAH dissipation rate and biological parameter concentrations were analyzed using Pearson correlation analysis. Clustering mean analysis was applied to identify and characterize various classes of treatment response patterns. To define the number of appropriate clusters for treatment responses based on explained variance, cluster analyses (K-means) were conducted with 2–5 clusters, and the amount of variation explained by the successive clusters was determined. An elbow plot of the explained variance of the clustering model as a function of the number of clusters k was made and three final clusters were selected. Based on these analyses, clusters with various degree of responsiveness to treatments were identified.
